# Video quality of nonalcoholic fatty liver disease on TikTok: A cross-sectional study

**DOI:** 10.1097/MD.0000000000039330

**Published:** 2024-08-23

**Authors:** Di Cheng, Kuiwu Ren, Xiang Gao, Kangkang Li, Panpan Wu, Rui Yang, Tao Cui, Kun Song, Jiangtao Yu

**Affiliations:** a Department of Hepato-Biliary-Pancreatic Surgery, Fuyang People’s Hospital of Anhui Medical University, Fuyang, Anhui Province, China; b Department of Hepato-Biliary-Pancreatic Surgery, Fuyang Hospital Affiliated Bengbu Medical College, Fuyang, Anhui Province, China.

**Keywords:** DISCERN, GQS, health information, NAFLD, social media, TikTok, video quality

## Abstract

The short-video application TikTok shows great potential for disseminating health information. We assessed the content, sources, and quality of information in videos related to nonalcoholic fatty liver disease (NAFLD) on TikTok. Our study aims to identify upload sources, content, and characteristic information for NAFLD videos on TikTok and further evaluate factors related to video quality. We investigated the top 100 videos related to NAFLD on TikTok and analyzed the upload sources, content, and characteristics of these videos. Evaluate video quality using the DISCERN tool and Global Quality Score (GQS). In addition, the correlation between video quality and video characteristics is further studied. In terms of video sources, the majority of NAFLD videos on TikTok (85/100, 85%) were posted by doctors, ensuring the professionalism of the content, and among the video content, disease knowledge was the most dominant video content, accounting for 57% (57/100) of all videos, and the average DISCERN and GQS scores of all 100 videos were 39.59 (SD 3.31) and 2.99 (SD 0.95), respectively. DISCERN and GQS data show that videos related to NAFLD do not have high-quality scores on TikTok, mainly fair (68/100, 68%) and moderate (49/100, 49%). In general, the quality of NAFLD video information from professional content and professional sources was higher than that of nonprofessional sources and nonprofessional content, the video quality of general surgeons was better than that of other department physicians, and the video quality of junior physicians was better than that of senior physicians. In terms of video correlation, durations, the number of fans, and the total number of works were negatively correlated with DISCERN scores (*R* < 0, *P* < .05), while likes, comments, collections, shares, and days since upload were not significantly correlated with DISCERN and GQS scores (*P* > .05). The medical information on TikTok is not rigorous enough to guide patients to make accurate judgments, platforms should monitor and guide publishers to help promote and disseminate quality content.

## 1. Introduction

Nonalcoholic fatty liver disease (NAFLD) is the most prevalent chronic liver disease worldwide, affecting nearly 25% of the general population and it predominantly affects obese and type 2 diabetes mellitus individuals^[1,2]^; NAFLD is estimated to affect about 75 to 100 million people (30–40% of adults) in the United States nearly, 25% of whom progress to nonalcoholic steatohepatitis, and an estimated 16 million (5% of the adult population).^[3,4]^ NAFLD ranges from a simple increase in lipid content within the liver (steatosis, NAFL to nonalcoholic steatohepatitis) with varying degrees of necrotizing inflammation, fibrosis, and eventually cirrhosis.^[5]^ The global prevalence of NAFLD is currently estimated to be as high as 1 billion people,^[6]^ and its prevalence is comparable to the prevalence of obesity. In recent years, the obesity rate in Asia has increased dramatically due to improved living standards and the influence of “westernized” diets, which has led to a rapid increase in the prevalence of NAFLD. Studies conducted in Korea, China, Japan, and Taiwan have reported prevalence rates of NAFLD ranging from 11% to 45%.^[7]^ This disease poses a significant economic and medical burden, given the large number of people affected and the associated complications.^[8]^ In the United States alone, the annual medical and social cost of NAFLD is estimated at US$ 292 billion,^[8]^ making it a major public health problem.

Nevertheless, there is evidence to suggest that improving diet quality^[9]^ and engaging in sustained or increased physical activity^[10–12]^ can reduce the risk of NAFLD, even in individuals with a high genetic risk.^[9]^ Primary care clinicians have a crucial role to play in promoting and coordinating lifestyle interventions, such as dietary modification and exercise, and in managing metabolic comorbidities.^[13]^

The Internet serves as a useful platform for the effective exchange of new information, and many new technology applications capitalize on this to educate patients about health-related topics, seeking web-based health information is also becoming increasingly popular, and many people rely on web-based resources to access health information and make medical decisions.^[14]^ TikTok is a short-video social media platform that has gained popularity among teenagers and young adults.^[15]^ It has nearly 800 million users and is viewed billions of times a day.^[16]^ On TikTok, users can create their own videos by lip-synching or dancing to popular songs, it offers rich forms of information, including text, images, audio, and video, and includes a large number of technical features such as comments, chats, follows, likes, and live streaming.^[17]^ These features make it easier for the general public to use TikTok as a source of health information. It is worth noting that the penetration and use of TikTok in some older age groups has also increased significantly.^[18]^ Given TikTok’s broad reach and better audience engagement than other traditional social media platforms,^[19]^ it has the potential to become a huge source of health information dissemination and grow in popularity among the average health consumer as an emerging source of health information.

While any emerging technology has good potential, patients may encounter a lot of issues when actually using technology, and assessing the quality of online health information sources is not an easy task for most laypeople.^[20,21]^ The quality of web-based health-related videos is also far from satisfactory. For example, more than a quarter of the most viewed COVID-19 videos on YouTube contain misleading information,^[22]^ 40% of cholelithiasis videos on TikTok had a DISCERN score below average, and video quality was negatively correlated with video likes and shares.^[23]^ These studies have shown that the overall quality of web-based videos with health information varies. Multiple previous studies have been conducted on the quality of videos about different diseases on TIKTOK. For example, COVID-19,^[24]^ diabetes,^[25]^ chronic obstructive pulmonary disease,^[26]^ cholelithiasis,^[23]^ and NAFLD.^[27]^ However, sources of high-quality video have not been adequately compared and the quality of videos of healthcare workers in different departments and titles has also not been adequately investigated. Therefore, the present study aims to categorize and compare videos related to NAFLD on TikTok in a more detailed manner, which will serve as a reference for publishers to disseminate higher-quality content on TikTok.

## 2. Methods

### 2.1. Data collection

In this cross-sectional study, a search was conducted on April 26, 2023 on the TikTok Chinese edition, and the search term used the Chinese term NAFLD, a total of 127 videos were screened, 11 duplicates were removed, 3 videos were not related to the theme, and the remaining 114 videos were selected, and we selected the top 100 Chinese videos for analysis and statistics until the top 100 Chinese videos were displayed. We limit our analysis to the top 100 videos in the first place because most average health consumers apply the “minimum effort” principle in online information acquisition activities, and they typically only look at the top search results rather than in-depth searches.^[28]^ Second, multiple studies have confirmed that videos other than the first 100 videos have no significant effect on the analysis.^[26,29]^ For each TikTok video about nonalcoholic fatty liver disease, the following characteristics are recorded and analyzed: title, number of likes, number of comments, number of collections, number of shares, tags, upload date, days since upload, video duration, total likes, total fans, content, and video source (uploader).

### 2.2. Classification of videos

Video sources are categorized as follows: (1) doctors, (2) individuals (i.e., nonmedical professionals), (3) news agencies (i.e., network media, newspapers, television stations, and radio stations), (4) organizations (i.e., hospitals, health authorities, research groups, universities, or colleges). The content was categorized as follows: (1) surgical operation, (2) disease information, (3) disease prevention, (4) disease reversion, and (5) case study. Most NAFLD can be reversed through lifestyle adjustments such as weight loss and bariatric surgery, and will not progress to more serious levels such as cirrhosis, and we classify them here as disease reversion for the treatment and medical advice of this population. This categorization allows us to group as many videos as possible with the same content, and to distinguish videos with different content. At the same time, our study subdivides medical professionals into 2 categories: (1) senior physicians (deputy director and above) and junior physicians (attending and below) and (2) general surgeons and other department physicians.

### 2.3. Quality assessment

The quality of the information in videos was assessed using the DISCERN instrument and Global Quality Scores (GQS). DISCERN is a short questionnaire designed to help health consumers and researchers assess the quality of health information (Table S1, Supplemental Digital Content, http://links.lww.com/MD/N437).^[30]^ It is one of the most widely used instruments for studying the quality of health information,^[31]^ has proven to be effective in the Chinese context,^[30]^ and has proven useful for assessing information quality on other video-based platforms such as YouTube,^[32]^ with 3 sections on the reliability of the publication (8 items), the quality of treatment option information (7 items), and the overall rating of the publication (1 item).^[33,34]^ Each of the 16 questions was scored on a scale of 1 (lowest score) to 5 (highest score), with overall DISCERN scores ranging from to very poor (16–26), poor (27–38), fair (39–50), good (51–62), and excellent (63–80).^[34,35]^ GQS assesses the value of education through 5 criteria (Table S2, Supplemental Digital Content, http://links.lww.com/MD/N439).^[36]^ The GQS score ranges from 1 to 5 points, with the highest score of 5 indicating high quality. All authors are practicing physicians working in hepatobiliary and pancreatic surgery with expertise in the diagnosis and treatment of diseases. During the screening and rating process, author CD and RKW simultaneously evaluated videos using the DISCERN instrument and GQS. If there is a disagreement between the 2 raters, the average of the ratings is taken to determine the final score, Use Microsoft Excel (2019) to extract, encode, and process statistics for each video.

### 2.4. Statistical analysis

Descriptive statistics were performed using means and SD for quantitative information, and qualitative information was demonstrated using percentages. Two-independent sample *t* test, one-way ANOVA, two-way ANOVA, and Tukey test were performed using Graphpad prism version 9.0 (Windows) (Graphpad software) to assess the significance of the difference. Pearson correlation analysis is used to assess relationships between quantitative variables, and *P* < .05 is considered statistically significant.

## 3. Results

### 3.1. Features of NAFLD videos

TikTok searched for the 100 videos it initially collected, with 722,941 likes, 21,764 comments, 82,832 collections, and 22,7251 shares. The average video duration is 65.8 (SD 64.78) seconds and the average number of total fans is 1050778.25 (SD 3646185.65). In addition, the average number of days after upload (days after upload) as of the date of data acquisition is 356.17 (SD 253.94) days. Table 1 shows the descriptive statistics of TikTok videos from different sources and content. According to video sources, 85% (85/100) of the videos were posted by doctors, the remaining sources were 5% (5/100) for individuals, 7% (7/100) for news agencies, and 3% (3/100) for organizations. Among the video content, disease information was the most dominant video content, accounting for 57% (57/100) of all videos. The remaining proportions were 14% (14/100) for surgical operation, 10% (10/100) for disease prevention, 13% (10/100) for disease reversion, and 6% (6/100) for case study.

**Table 1:** Descriptive statistics for TikTok videos of different sources and content.

Variables	Likes, mean (SD)	Comments, mean	Collections, mean	Shares, mean	Days since upload (days)	Durations (seconds)	Fans	Number of works
		(SD)	(SD)	(SD)	Mean (SD)	Mean (SD)	Mean (SD)	Mean (SD)
Video source (n = 100)								
** **Doctors (n = 85)	7014.40 (41549.23)	212.47 (903.36)	758.54 (3981.48)	2502.90 (13874.92)	332.07 (218.04)	55.93 (50.93)	697210.56 (2828764.15)	817.98 (989.33)
* *Senior (n = 64)	2517.39 (6087.77)	123.52 (340.57)	286.17 (606.83)	891.33 (1982.80)	334.02 (210.12)	50.70 (48.18)	341681.94 (544870.67)	961.11 (1090.08)
* *Junior (n = 21)	20719.57 (82897.17)	483.57 (1720.35)	2198.14 (7907.54)	7414.38 (27613.99)	326.14 (246.09)	71.86 (56.84)	1780708.05 (5570968.45)	381.76 (320.07)
** **General surgery (n = 48)	1690.44 (3739.01)	117.96 (363.08)	281.75 (612.50)	910.31 (2116.27)	339.13 (188.38)	40.04 (27.64)	302965 (387593.28)	1123.06 (1158.50)
** **Others (n = 37)	13921.16 (62634.25)	335.08 (1305.63)	1377.08 (5983.51)	4568.97 (20870.60)	322.92 (253.87)	76.54 (65.44)	1208664.24 (4242510.24)	422.19 (496.46)
Individuals (n = 5)	21734.8 (29454.84)	636.6 (883.15)	3396.4 (5612.55)	7060.2 (9693.34)	310 (190.95)	112.6 (44.14)	1167404.6 (1471814.58)	436.8 (371.04)
** **News agencies (n = 7)	2539.71 (2418.12)	70.43 (69.21)	192.43 (149.30)	1306.14 (1470.51)	691.43 (473.38)	130.43 (143.27)	5675285.71 (8870676.44)	6118 (8842.20)
** **Organizations (n = 3)	88.33 (122.02)	9.33 (11.02)	9 (9.64)	20 (25.12)	333.67 (160.95)	116.67 (72.14)	83635 (75318.06)	394 (251.54)
Video content (n = 100)								
** **Surgical operation (n = 13)	1810 (4578.76)	222.69 (602.75)	113.62 (178.59)	806.46 (1874.34)	322.15 (139.29)	28.38 (10.02)	372158.4 (290036.44)	1930.08 (1233.44)
** **Disease knowledge (n = 57)	10105.33 (50847.90)	265.72 (1078.60)	961.18 (4782.90)	3451.44 (16922.07)	355.61 (237.04)	66.82 (59.64)	1187414.46 (4517859.77)	1080.49 (3191.65)
** **Disease prevention (n = 10)	3569.5 (10347.81)	144.8 (398.22)	816.6 (2297.48)	1459.6 (3730.36)	261.4 (153.56)	72.6 (61.62)	1062914.2 (3208410.20)	335.3 (289.27)
** **Disease reversion (n = 14)	6039.36 (14141.13)	143 (336.25)	1281.57 (3431.30)	2370.36 (5264.36)	455.21 (430.33)	95.79 (103.32)	929714.29 (1544722.6)	463.14 (451.15)
** **Case study (n = 6)	526.83 (405.13)	45.5 (50.83)	76.67 (76.26)	375.67 (583.03)	362 (169)	55.83 (45.29)	1485333.33 (2831527.76)	3200.67 (4463.83)

SD = standard deviation.

### 3.2. Video quality assessments

**Figure 1.**
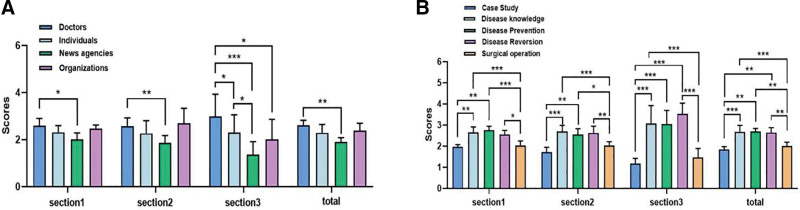
(A) DISCERN scores for TikTok videos of different sources. (B) DISCERN scores for TikTok videos of different contents (*P < .05, **P < .01, ***P < .001).

The average DISCERN and GQS scores for all 100 videos were 39.59 (SD 3.31) and 2.99 (SD 0.95). We used the DISCERN score to compare video quality based on different sources and content, and the 3 sections and the overall score of DISCERN were analyzed. In section 1, doctors scored higher DISCERN scores than news agencies (*P* < .05) and disease information and disease prevention DISCERN scores significantly higher than surgical operation and case study (*P* < .001 and *P* < .01, respectively). NAFLD videos from professional sources and content are more reliable than videos from nonprofessional sources and content. In section 2, doctors scored higher DISCERN scores than news agencies(*P* < .01), disease information DISCERN scores significantly higher than surgical operation and case study(*P* < .001). The results of the study showed that videos of NAFLD from professional sources and content were better than videos from nonprofessional sources and content in providing treatment information. In section 3, DISCERN score of doctors were significantly higher than news agencies (*P* < .0001), and disease information, disease reversion, and disease prevention DISCERN scores were significantly higher than surgical operation and case study(*P* < .001). Overall, the overall score of doctors was significantly higher than that of news agencies (*P* < .01), and disease information and disease prevention were significantly higher than that of surgical operation and case study (*P* < .001 and *P* < .01, respectively). In general, the quality of NAFLD video information from professional content and professional sources is higher than that of nonprofessional sources and nonprofessional content (Fig. 1A and B).

**Figure 2.**
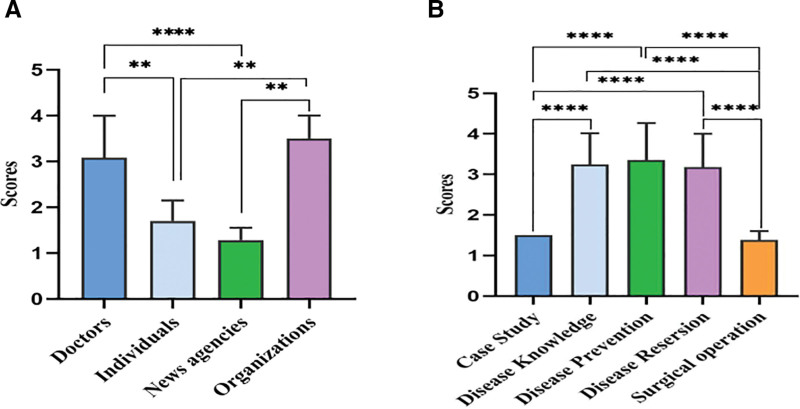
(A) Global Quality Scores analysis for TikTok videos of different sources. (B) Global Quality Scores analysis for TikTok videos of different content (*P < .05, **P < .01, ***P < .001).

**Figure 3.**
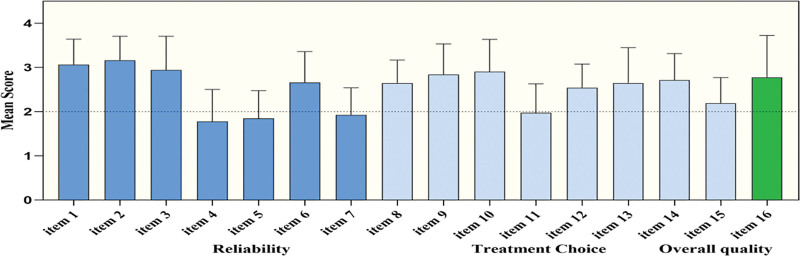
The mean DISCERN score for each item.

In GQS analysis, doctors and organizations scored significantly higher GQS scores than news agencies (*P* < .001 and *P* < .01, respectively), and GQS scores for disease information, disease reversion, and disease prevention were significantly higher than those for surgical operation and case study (*P* < .001) (Fig. 2A and B). DISCERN and GQS data show that NAFLD videos do not have high quality scores on TikTok, mainly fair (68/100, 68%) and moderate (49/100, 49%) (Table 2). The average DISCERN score for each item is shown in (Fig. 3). The main reasons for the low rating are that the introductory statement is not supported by the evidence-based sources cited (question 4) and the timeliness of the knowledge content is not clear (question 5).

**Table 2:** The 5-level scores of DISCERN and Global Quality Scores (GQS; n = 100).

Scores	Value, n (%)
DISCERN	
16–26 (very poor)	0 (0)
26–38 (poor)	31 (31)
38–50 (fair)	68 (68)
50–62 (good)	1 (1)
62–80 (excellent)	0 (0)
GQS score	
1 (poor)	3 (3)
2 (general poor)	26 (26)
3 (moderate)	49 (49)
4 (good)	18 (18)
5 (excellent)	4 (4)

GQS = Global Quality Score.

**Figure 4.**
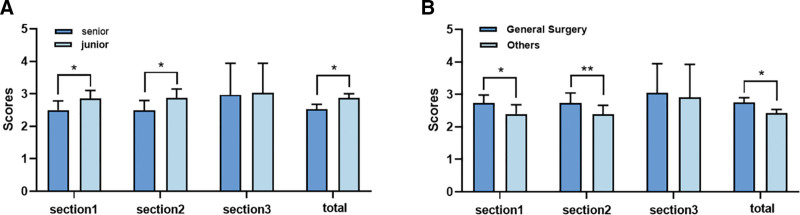
(A) DISCERN scores for TikTok videos of different seniority of doctors. (B) DISCERN scores for TikTok videos of different departments of doctors (*P < .05, **P < .01, ***P < .001).

**Figure 5.**
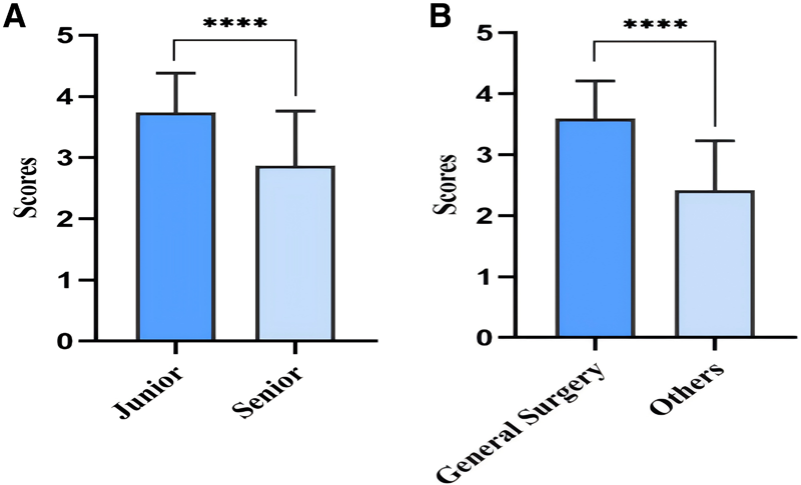
(A) Global Quality Scores analysis for TikTok videos of different seniority of doctors. (B) Global Quality Scores analysis for TikTok videos of different departments of doctors.

In DISCERN scores, general surgeons outperformed other specialists in video reliability and video information provision (*P* < .05 and *P* < .01, respectively). Among them, junior physicians slightly outperformed senior physicians in video reliability and provision of video information (*P* < .05) (Fig. 4A and B); in the GQS analysis, general surgeons had significantly higher GQS scores than physicians in other specialties (*P* < .001). Among them, junior physicians had significantly higher GQS scores than senior physicians (*P* < .001) (Fig. 5A and B).

### 3.3. Correlation analysis

Spearman correlation analysis showed positive correlations for the following variables: likes and comments (*R* = 0.96, *P* < .0001), likes and collections (*R* = 0.97, *P* < .0001), likes and shares (*R* = 0.99, *P* < .0001), comments and collections (*R* = 0.93, *P* *≤* .0001), comments and shares (*R* = 0.96, *P* < .0001), collections and shares (*R* = 0.97, *P* *≤* .0001). In addition, the number of fans was positively correlated with likes, comments, collections, and shares (*R* = 0.66, *R* = 0.64, *R* = 0.66, *R* = 0.66, *P* < .0001, respectively), and the total number of works and fans were also positively correlated (*R* = 0.66, *P* < .0001) (Table 3). The DISCERN score was negatively correlated with video duration, fans, and total number of works (*r* = −0.31, *P* < .0001; *r* = −0.21, *P* = .03, *r* = −0.26, *P* < .01, respectively) (Table 4).

**Table 3:** The relationship level between video variables.

Variable and analysis*	Likes	Comments	Collections	Shares	Days since upload	Durations	Fans	Number of works
Likes								
*R* value	1	–†	–	–	–	–	–	–
*P* value	–	–	–	–	–	–	–	–
Comments								
*R* value	0.96	1	–	–	–	–	–	–
*P* value	<.0001‡	–	–	–	–	–	–	–
Collections								
*R* value	0.97	0.93	1	–	–	–	–	–
*P* value	<.0001‡	<.0001‡	–	–	–	–	–	–
Shares								
*R* value	0.99	0.96	0.97	1	–	–	–	–
*P* value	<.0001‡	<.0001‡	<.0001‡	–	–	–	–	–
Days since upload								
*R* value	0.08	0.09	0.02	0.07	1	–	–	–
*P* value	.45	.39	.87	.5	–	–	–	–
Durations								
*R* value	0.16	0.16	0.18	0.17	0.11	1	–	–
*P* value	.10	.12	.07	.10	.3	–	–	–
Fans								
*R* value	0.66	0.64	0.66	0.66	−0.01	0.66	1	–
*P* value	<.0001‡	<.0001‡	<.0001‡	<.0001‡	.9	.38	–	–
Number of works								
*R* value	0.01	0.03	0.00	0.01	−0.05	−0.11	0.66	1
*P* value	.94	.74	.98	.91	.63	.26	<.0001‡	–

* Pearson correlation analysis and *r* correlation coefficient.

† Not applicable.

‡ *P* < .0001.

**Table 4:** Pearson correlation analysis between video quality scores and video variables.

Variable and analysis	DISCERN	GQS
Likes		
*R* value	−0.01	0.02
*P* value	.95	.86
Comments		
*R* value	0.00	0.02
*P* value	.97	.87
Collection		
*R* value	0.01	−0.04
*P* value	.96	.69
Shares		
*R* value	0.00	0.00
*P* value	.98	.96
Days since upload		
*R* value	−0.13	−0.04
*P* value	.20	.72
Durations		
*R* value	−0.31	−0.18
*P* value	<.0001*	.08
Fans		
*R* value	−0.21	−0.18
*P* value	.03**	.08
Numbers of works		
*R* value	−0.26	−0.19
*P* value	.01**	.06

GQS = Global Quality Scores.

* *P* < .0001.

** *P* < .05.

## 4. Discussion

### 4.1. Principal findings

In this cross-sectional study, we analyzed characteristic information about NAFLD videos on TikTok at a single time point and evaluated video quality using the DISCERN and GQS instruments. The majority of NAFLD videos on TikTok (85/100, 85%) were posted by doctors, ensuring the professionalism of the content. However, according to the DISCERN and GQS video quality ratings, the quality of the doctors’ videos was not as good as expected. It is worth noting that general surgeons had better video quality than physicians from other departments, and the seniority of the doctor did not improve the video quality. The number of likes, comments, collections, and shares partly reflects the popularity of a video or post.^[37,38]^ Our study found a positive correlation between likes, comments, collections, and shares, with bloggers who have more total fans receiving more comments and retweets, this indicates that bloggers with more fans are more likely to attract traffic, and popular videos are more likely to become even more popular. The number of comments is also positively correlated with the number of collections and shares, suggesting that videos with more comments are more likely to be collected and shared, which is consistent with the findings of previous studies.^[23]^ Additionally, the number of days after uploading is positively correlated with likes and shares, indicating that videos gain more exposure over time, leading to more opportunities to gain fans.

### 4.2. The overall quality of the video

The average DISCERN and GQS scores for the 100 NAFLD videos on TikTok were 39.59 (SD 3.31) and 2.99 (SD 0.95), respectively, indicating that most of the videos were rated as moderate or poor in quality. This is consistent with a previous study that evaluated 100 videos on gallstone disease, which reported average DISCERN and GQS scores of 39.61 (SD 11.36) and 2.76 (SD 0.95),^[23]^ respectively. However, our study found that longer video duration was not associated with better video quality, which contrasts with previous findings. We speculate that this could be due to several factors. Firstly, the DISCERN scale may not be directly comparable across different disease categories, and different evaluators may use different scoring criteria, introducing potential biases. Secondly, our analysis showed that the average video duration on TikTok was 65.8 seconds, which is longer than in previous studies. This may lead to visual fatigue and reduce the overall production quality of the video, as well as increasing the amount of redundant or irrelevant information that does not contribute to video quality.

### 4.3. The correlation between video quality and video characteristics

Table 4 shows that the days since upload, number of fans, and total number of works are negatively correlated with DISCERN and GQS scores, while the correlation between likes, comments, collections, and shares and DISCERN and GQS scores is not significant. This suggests that video popularity does not necessarily reflect video quality, and that videos published by high-profile traffic bloggers with more followers may have lower quality. Longer video duration and more frequent posting may increase the exposure of these videos, but their quality does not match their visibility, leading to a larger number of viewers receiving lower quality content. The spread of low-quality videos can still attract high click-through rates, likes, and shares, which can be amplified by TikTok’s recommendation algorithm that favors popular videos. Social media companies aim to capture users’ attention by creating highly immersive and engaging services,^[39]^ prioritizing entertaining images and videos. However, educational videos about medical topics may be perceived as less engaging or boring by users, leading to less popularity compared to more entertaining content.

### 4.4. Possible interventions

Our study found that general surgeons had higher-quality videos than other department physicians, and junior physicians had higher-quality videos than senior physicians. This suggests that more professional creators may publish medical knowledge that is more authoritative and reliable, while seniority does not necessarily lead to higher video quality. Younger doctors may have a better understanding of new media trends and be more adept at creating high-quality content. Additionally, their video explanations may be more dynamic and engaging compared to the more static and dull videos produced by older doctors. However, senior physicians have more experience and professional expertise, and should be supported and trained by the platform to improve their video production skills. For example, they could add trending labels and background sounds, use rich complementary visual effects such as vivid images or realistic characters, and avoid redundancy and boredom in their video content. Furthermore, certified specialists’ popular science content should be recommended first to ensure high-quality content is prioritized. Our research also identified a pattern of bloggers repeatedly posting multiple videos of the same category, with the same content and narrative technique. This strategy may attract more views and attention, helping them accumulate fans more quickly, but can lead to poor video quality due to a lack of diversity and originality. TikTok provides live streaming tips and traffic monetization functions, allowing popular bloggers to profit from their high exposure and popularity. However, TikTok should strengthen its censorship and supervision of bloggers who publish duplicate videos to prevent the malignant attraction of traffic and fans.

## 5. Conclusion

Videos on TikTok about NAFLD are mainly provided by physicians and are of average overall quality, which does not guide patients to make accurate judgments. Influential bloggers have a responsibility to create more comprehensive and accurate content, and platforms should strengthen regulations to meet the public’s information needs. Highly qualified health professionals should create more engaging educational videos and improve their communication skills to effectively disseminate high-quality content.

## Author contributions

**Conceptualization:** Di Cheng.

**Data curation:** Di Cheng, Xiang Gao, Kangkang Li, Tao Cui.

**Formal analysis:** Panpan Wu, Rui Yang, Kun Song.

**Methodology:** Di Cheng, Kuiwu Ren.

**Writing – original draft:** Di Cheng.

**Writing – review & editing:** Jiangtao Yu.

## Supplementary Material
