# Supplementary material for: Video quality of nonalcoholic fatty liver disease on TikTok: A cross-sectional study
Source: Medicine (Baltimore). 2024 Aug 23;103(34):e39330. doi: 10.1097/MD.0000000000039330 (PMC11346868; doi:10.1097/MD.0000000000039330)
Supplement: Supplementary file 2 [file medi-103-e39330-s002.docx]

Supplemental Digital Content 2

*GQS Description*

1 Poor quality; poor flow of the site; most information missing; not at all useful for patients

2 Generally poor quality and poor flow; some information listed but many important topics missing; of very limited use to patients

3 Moderate quality; suboptimal flow; some important information is adequately discussed but others poorly discussed; somewhat useful for patients

4 Good quality and generally good flow; most of the relevant information is listed, but some topics not covered; useful for patients

5 Excellent quality and excellent flow; very useful for patients

*GQS* = Global Quality Score.
